# Linear-Shaped Low-Bandgap Asymmetric Conjugated Donor Molecule for Fabrication of Bulk Heterojunction Small-Molecule Organic Solar Cells

**DOI:** 10.3390/molecules28041538

**Published:** 2023-02-05

**Authors:** Abdullah, Sei-Jin Lee, Jong Bae Park, Yang Soo Kim, Hyung-Shik Shin, Ashique Kotta, Qamar Tabrez Siddiqui, Youn-Sik Lee, Hyung-Kee Seo

**Affiliations:** 1 Korea Basic Science Institute (KBSI), Jeonju Center, Jeonju 54896, Republic of Korea; 2 Korea Basic Science Institute (KBSI), Daejeon 34133, Republic of Korea; 3 School of Chemical Engineering, Jeonbuk National University, Jeonju 54896, Republic of Korea; 4 Future Energy Convergence Core Center, Jeonbuk National University, Jeonju 54896, Republic of Korea

**Keywords:** hexyl bithiophene, electron donor, thermal stability, low bandgap, organic solar cell

## Abstract

A linear–shaped small organic molecule (E)-4-(5-(3,5-dimethoxy-styryl)thiophen-2-yl)-7-(5″-hexyl-[2,2′:5′,2″-terthiophen]-5-yl)benzo[c][1,2,5]thiadiazole (MBTR) comprising a benzothiadiazole (BTD) acceptor linked with the terminal donors bithiophene and dimethoxy vinylbenzene through a π-bridge thiophene was synthesized and analyzed. The MBTR efficiently tuned the thermal, absorption, and emission characteristics to enhance the molecular packing and aggregation behaviors in the solid state. The obtained optical bandgap of 1.86 eV and low-lying highest occupied molecular orbital (HOMO) level of −5.42 eV efficiently lowered the energy losses in the fabricated devices, thereby achieving enhanced photovoltaic performances. The optimized MBTR:PC_71_BM (1:2.5 *w/w*%) fullerene-based devices showed a maximum power conversion efficiency (PCE) of 7.05%, with an open-circuit voltage (V_OC_) of 0.943 V, short-circuit current density (J_SC_) of 12.63 mA/cm^2^, and fill factor (FF) of 59.2%. With the addition of 3% 1,8-diiodooctane (DIO), the PCE improved to 8.76% with a high V_OC_ of 1.02 V, J_SC_ of 13.78 mA/cm^2^, and FF of 62.3%, which are associated with improved charge transport at the donor/acceptor interfaces owing to the fibrous active layer morphology and favorable phase separation. These results demonstrate that the introduction of suitable donor/acceptor groups in molecular design and device engineering is an effective approach to enhancing the photovoltaic performances of organic solar cells.

## 1. Introduction

Rapidly growing bulk heterojunction (BHJ) organic solar cells (OSCs) show promising application potential for high energy throughput owing to their distinguished features, such as fixed molecular weight, mechanical flexibility, optical transparency, and solution processability [1,2,3,4,5,6,7]. Over the past few years, BHJ OSCs containing small molecules have achieved exciting progress while benefitting from the development of new photoactive materials and device structure engineering approaches, with power conversion efficiencies (PCEs) exceeding 18% for non-fullerene acceptor (NFA)-based OSCs [8,9,10,11]. Compared to the NFAs, PCEs of approximately 11% have been achieved for traditional OSCs using fullerene derivatives [12]. Over time, fullerene derivatives such as phenyl-C_61_-butyric acid methyl ester (PC_61_BM, PC_71_BM) have been widely used as electron acceptors to construct BHJ OSCs. In OSCs, the fullerene PCBM is an important class of electron acceptors because of its potential characteristics of high electron affinity, increased carrier mobility, and ability to form evenly distributed surfaces for efficient electron transport [13]. Gupta et al. recently developed efficient donor molecules containing donor–acceptor–donor (D–A–D) units that exhibited a PCE of 8.00% when blended with a PCBM acceptor [14]. Abdullah et al. reported a reasonable PCE of 6.88% using a simple benzoselenadiazole containing small molecules and systematic optimization of the end groups with different electron donor units, such as triphenylamine (TPA) and alkyl bithiophene [15]. The π–conjugated system of small molecules efficiently tunes the photophysical properties while lowering the highest occupied molecular orbital (HOMO) and lowest unoccupied molecular orbital (LUMO) [16,17,18]. The selection of an appropriate donor–acceptor unit for developing small molecules is very important in the design of semiconducting materials for achieving high electrical properties [19]. From the molecular design perspective, the optical and energy levels of conjugate molecules can be realistically tuned by substituting different electron donating/accepting groups, such as alkylated bithiophene, alkyl phenyl, and heterocyclic units, in the donor/acceptor backbone [20,21,22,23,24]. The heterocyclic benzothiadiazole (BTD) is believed to be a useful building block in the design of organic electronic materials owing to its higher electron-withdrawing capability, electron affinity (EA), and high stability [16,17,18,19]. Alternatively, two rigid nitrogen atoms in the BTD ring form hydrogen bonds with the adjacent atoms to improve the molecular backbone’s planarity [25]. Many BTD-containing polymers and small molecules have been widely used in several organic electronic applications, such as solar cells, organic light-emitting diodes (OLEDs), and organic field-effect transistors (OFETs) [26,27,28]. However, thiophene and oligothiophene derivates have been extensively studied as donor units for developing small organic molecules owing to their ease of synthesis, superb stability, and excellent hole/electron transport characteristics [29,30,31]. In particular, oligothiophene is proven to be efficient in the design of D–A–D conjugated molecules along with an electron-deficient acceptor unit, since these molecules increase the light-harvesting ability through intramolecular charge transfer [32]. It is observed from the literature that hexyl bithiophene has been introduced as the end-group units in BTD-containing derivatives to extend the conjugation length through interchromic π–π stacking and desirable film-forming capabilities to produce photoactive layers with nano morphologies [33]. In some of our recently reported work, a simple BTD-based small molecule having the D_1_–π–A–D_2_ configuration with a methoxy-substituted vinylbenzene or styryl and hexyl bithiophene end-capped unit was designed by Lamiaa et al. It demonstrated excellent optoelectronic properties and achieved a maximum PCE of 3.53% with an improved fill factor (FF) in BHJ devices [34]. In another work, a planar D-π-A-type vinylbenzene-based small molecule was designed that efficiently tuned optical and electrochemical properties and realized different H- and J-aggregation in the solid state [35]. The designed molecule was utilized as donor material in fullerene-based BHJ OSCs, delivering a PCE of 4.05% with extended J_sc_ of 10.43 mA/cm^2^. It has been reported that the incorporation of electron-rich thiophene and its derivative as the π-bridge between the donor and acceptor units not only extends the conjugation length and π–π stacking ability, but also broadens the absorption band and molecular coplanarity while enhancing charge mobility [36]. Therefore, it is necessary to achieve a subtle balance among molecular design, energy levels, film morphology, and engineering in device fabrication to realize high-performance OSCs [37]. One of the well-known challenges in OSCs is to maintain open-circuit voltage (V_OC_) while improving the short-circuit current density (J_SC_) and fill factor (FF) value, which could be attained through better molecular design, desired film morphology, and controlled device structure.

Inspired by the above discussed photophysical and electronoic properties of BTD, thiophene and its derivatives, and vinyl benbenzene, the present work focuses on the synthesis of a new small molecule (E)-4-(5-(3,5-dimethoxy-styryl)thiophen-2-yl)-7-(5″-h-exyl-[2,2′:5′,2″-terthiophen]-5-yl)benzo[c][1,2,5]thiadiazole (MBTR) in a stepwise synthetic pathway using BTD as the central core unit that is end-capped with different donor hexyl bithiophene and methoxy substituted moieties connected through the π-bridge thiophene unit. The synthesized MBTR is applied as a donor molecule with a fullerene acceptor (PC_71_BM) for the fabrication of BHJ OSCs with or without a 1,8-diiodooctane (DIO) additive. The highest PCE of ~8.76% was achieved for the best device, MBTR:PC_71_BM (1:2.5 *w/w*%, 3% DIO), with an improved V_OC_ of ~1.021 V, controlled J_SC_ of 13.78 mA/cm^2^, and FF of ~62.3%.

## 2. Results and Discussion

### 2.1. Synthesis of Small Molecule and Thermal Properties

**Scheme 1:**
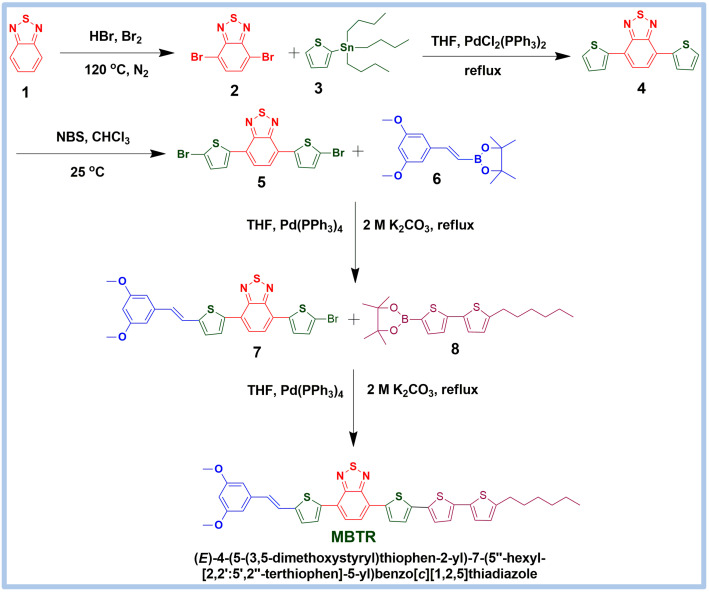
Synthesis protocols for the intermediates and MBTR molecule.

**Figure 1:**
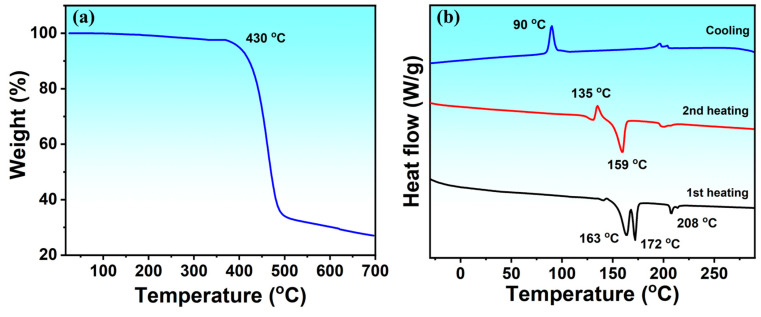
(**a**) TGA and (**b**) DSC results of the MBTR molecule.

A schematic of the synthesis is illustrated in Scheme 1, and the detailed methods used are discussed in the experimental part. The novel MBTR molecule was synthesized using commercial BTD as the main reactant and compound 3 as the π-bridge unit. Subsequently, monomer 7 was prepared using compounds 5 and 6 by the Suzuki coupling reaction, which was further used with compound 8 to obtain the MBTR small molecule with a yield of 58% [38,39]. The intermediate and target molecules were characterized by nuclear magnetic resonance (NMR), Fourier-transform infrared (FTIR), and mass spectroscopy to confirm the chemical structure shown in the Supplementary Materials (Figures S1–S3). Further, the thermal properties of MBTR were detected by thermogravimetric analysis (TGA) and differential scanning calorimetry (DSC), as displayed in Figure 1a,b. The TGA thermogram indicated a high thermal stability at 430 °C with less than 5% weight loss, and the DSC plot exhibited multiple endothermic melting transition crystallization transitions attributed to the high thermal stability and crystallinity, which meet the desired requirements for organic photovoltaic (PV) device fabrication.

### 2.2. Optical, Electrochemical, and Emission Investigations of MBTR

**Figure 2:**
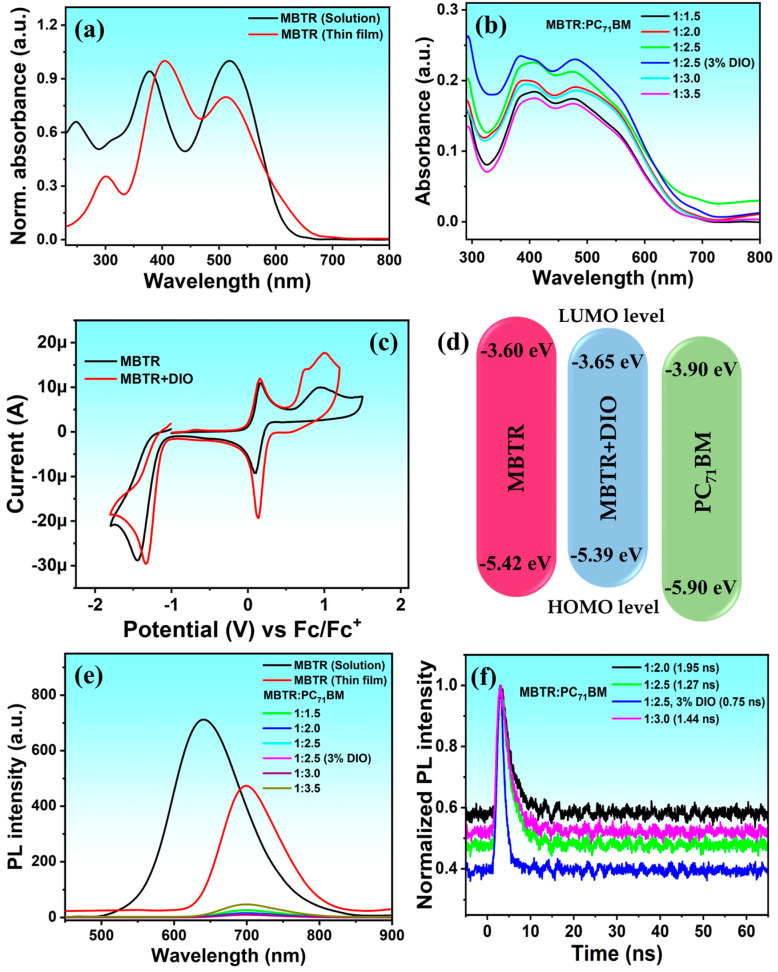
Optoelectronic properties of the MBTR molecule: (**a**,**b**) UV−visible absorption spectra; (**c**,**d**) electrochemical cyclic voltammogram and energy level diagram; (**e**) PL emission spectra; and (**f**) TRPL spectra of MBTR:PC_71_BM blend films.

The optical properties of the MBTR molecule in solution, solid-state thin film, and blend films were characterized via UV-visible spectroscopy, as shown in Figure 2a,b; the corresponding data are summarized in Table 1. The MBTR in the solution form displayed two absorption peaks (λ_max_) located at 378 and 519 nm, with the high-lying region assigned to the π–π* electronic transition and low-lying region resulting from intramolecular charge transfer transition [40]. The high-lying absorption band in the thin film was observed at 406 nm owing to the strong intermolecular π–π* interactions, and a negligible blue shift was observed in the low-lying region centered at 515 nm due to H-aggregation in the solid state [35]. Eventually, the absorption coefficient for the thin film was evaluated as ~4.01 × 10^4^ M^−1^ cm^−1^. In contrast to the solution, the thin-film absorption covers a wide region in the UV-visible spectrum with a narrow optical bandgap (E_g_) of approximately 1.86 eV. Analogous to other small molecules, the narrow E_g_ of the MBTR molecule indicates its more efficient intermolecular charge transfer (ICT) interactions, and it is believed that a narrow optical bandgap is related to low energy losses in PV devices, which is beneficial for improving the V_OC_ [41]. As shown in Figure 2b, the active layer blend film loaded with different weight amounts of the MBTR and PC_71_BM acceptor exhibits absorption in the range of 320–670 nm. However, the blend film with MBTR:PC_71_BM (1:2.5 *w/w*%) loaded with 3% DIO shows a higher absorption intensity than other blend films. Thus, the blend film of MBTR:PC_71_BM (1:2.5 *w/w*%) with 3% DIO significantly enhanced photon-harvesting properties, which may have a great influence on generating a higher photocurrent (J_SC_) [42].

The electrochemical behavior of the MBTR molecule was measured by cyclic voltammetry (CV) to extract the frontier energy levels. The oxidation (+0.62 and +0.59 V) and reduction (−1.20 and −1.15 V) potentials were extracted from the onset of the CV graph against ferrocene/ferrocenium (0.43 V). The HOMO and LUMO energy levels for MBTR without and with DIO were calculated as −5.42/−3.60 eV and −5.39/−3.65 eV, respectively, from the onset of oxidation and reduction by assuming the standard energy level of Fc/Fc^+^ (4.8 eV) below vacuum. The charge transfer at the active layer interface depends on the HOMO–HOMO and LUMO–LUMO offset values between the utilized donor and acceptor materials [43]. Effective electron transfer occurs at the donor/acceptor (D/A) interface from the MBTR donor to the PC_71_BM acceptor owing to a sufficient driving force of 0.30 (without DIO) and 0.25 eV (with DIO) between the donor and acceptor LUMO levels for separation of the coulombic-bonded excitons into free charge carriers [44]. Furthermore, the V_OC_ of the solar device is proportional to the difference between the donor HOMO and acceptor LUMO levels [45]. Therefore, a donor with a deep-lying HOMO level and large energy gap significantly results in a device with high V_OC_.

To understand the excitation energy transfer between the D/A of the photoactive layer, the photoluminescence (PL) emission spectra were measured. The output of the PL measurement is shown in Figure 2e. The MBTR PL spectrum was obtained with excitation at 450 nm and exhibited an intense and broad emission band (λ_max_) at 640 nm in the solution state. Next, the MBTR thin film excited at same excitation wavelength (450 nm) displayed a prominent red-shift in the emission spectrum at 700 nm because of J-type aggregation-induced emissions and high energy transfer in the D/A groups [46]. The photoactive blend films of optimized MBTR:PC_71_BM (1:2.5 *w/w*%) without and with DIO showed noticeable quenching effects of approximately 97% and 99%, respectively, against the pure MBTR film, indicating efficient photoinduced charge transfer (electron and hole) driven by the MBTR donor molecule and PC_71_BM acceptor [47]. However, the low quenching (90–94%) observed in the 1:1.5 and 1:3.5 (*w/w*%) ratios indicate limited electron and hole transfer from the donor to acceptor, which could explain the poor performance of PV devices [48]. The fluorescence lifetime of the photoactive MBTR:PC_71_BM film presented in Figure 2f was inspected by time-resolved photoluminescence (TRPL) measurement, which further verified the efficiency and charge transfer dynamics of the fabricated devices. It is evident from the TRPL results that the MBTR:PC_71_BM (1:2.5) blend film exhibits a faster decay lifetime of ~1.27 ns versus the other blend films, leading to well-organized photoexcited charge separation. Compared to the pristine MBTR:PC_71_BM (1:2.5), the blend film of MBTR:PC_71_BM (1:2.5) loaded with 3% DIO displays an even faster decay of ~0.75 ns, which is in line with the PL quenching effect [49,50]. The faster fluorescence decay implies more efficient exciton dissociations, yielding lower charge recombinations in PV devices that are consistent with the improved PV parameters.

### 2.3. Photovoltaic Characteristics of MBTR

**Figure 3:**
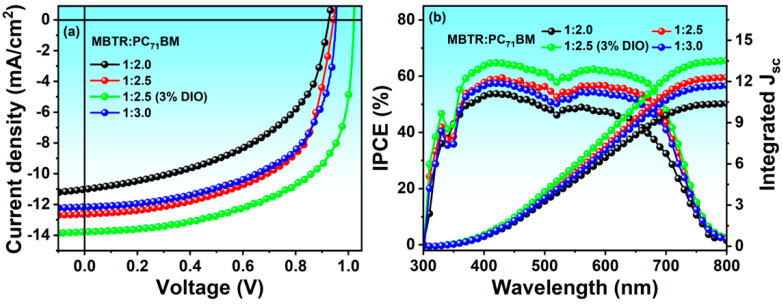
(**a**) J−V and (**b**) IPCE characteristics of the MBTR:PC_71_BM BHJ OSCs.

The PV performances of BHJ devices fabricated with the electron donor MBTR and electron acceptor PC_71_BM with a ITO/PEDOT:PSS/photoactive layer/Au structure were investigated using the current density vs. voltage (J–V) measurements. The J–V curves of the PV devices are shown in Figure 3, and the detailed data are summarized in Table 2. The OSCs displayed a low PCE of 4.24% with a V_OC_ of 0.910 V, J_SC_ of 10.36 mA/cm^2^, and FF of 45.0% for MBTR:PC_71_BM (1:1.5 *w/w*%). After changing the D/A content in the blend ratio (i.e., MBTR:PC_71_BM at 1:2.0 *w/w*%), the performances of the resulting devices gradually improved to a PCE of approximately 5.15%, and there were significant improvements in the other PV parameters, such as the J_SC_, V_OC_, and FF values. The PCE of the optimized devices reached up to 7.05% with a loaded content of MBTR:PC_71_BM (1:2.5 *w/w*%) and an enhanced V_OC_ of 0.943 V, J_SC_ of 12.63 mA/cm^2^, and FF of 59.2%. Further, the PV parameters improved with the addition of 3% DIO in the photoactive blend of BHJ OSCs to produce a high J_SC_ of 13.78 mA/cm^2^, V_OC_ of 1.02 V, and increased FF of 62.3%, which together delivered a maximum PCE of 8.76%. Upon the addition of DIO, the interfacial area between the donor and acceptor was enhanced, and photo exciton charge dissociation was facilitated, producing an enhanced V_OC_ [51]. In addition, the high V_OC_ values in devices processed with 3% DIO are attributed to the low energy offset of about 0.51 eV between the HOMO of MBTR and PC_71_BM, which also lowers the energy loss to attain a high V_OC_ in the OSCs [52]. The energy loss of the device estimated using the equation E_loss_ = E_g_^opt^ − qV_OC_ is 0.84 V compared to the value of 0.92 V for devices without DIO, which is consistent with obtained V_OC_ value, as illustrated in Table 2. Further, the improvement in J_SC_ is related to the superior charge splitting of the photogenerated carriers and their transport in the BHJ devices as a result of the active layer intermixing morphology and appropriate crystallinity that were observed after adding DIO [53]. In addition, the decrease in series resistance (R_S_) and increase in shunt resistance (R_Sh_) values are proportionally related for improving the FF of the OSCs. The R_S_ value of the MBTR:PC_71_BM (1:2.5 *w/w*%) loaded with 3% DIO gradually declined to 1.20 Ω cm^2^, and the R_Sh_ value increased to 69.78 Ω cm^2^ compared with the R_S_ of 1.59 Ω cm^2^ and R_Sh_ of 60.50 Ω cm^2^ for the optimized devices without DIO. Such resistance values are assumed to decrease molecular recombination and thereby afford high carrier mobility to enable a percolation passage for adequate transport of the dissociated electrons and holes in PV cells; hence, a high FF is noted compared to cells fabricated without DIO [54]. The accuracy of FF for organic devices was investigated by the fourth quadrant I-V characteristics of MBTR:PC_71_BM with the varying donor: acceptor (*w/w*% ratio) content under the illumination of 1.5 A.M. at 100 mW/cm^2^, which is explained in the supporting information (Figure S3).

The photoresponses of solar devices are expressed by the incident photon-to-current conversion efficiency (IPCE) measurements. As shown in Figure 3, the IPCE spectra exhibit photoresponses in the 300–800 nm range and cover 50–70% of the IPCE region. From the integration of the IPCE spectra, the integrated J_SC_ values are estimated as 12.29, 13.51, and 11.70 mA/cm^2^ for the MBTR:PC_71_BM (1:2.5 without and with DIO as well as 1:3.0 *w/w*%), which are consistent with the J_SC_ values extracted from the J–V curve. Further, we investigated the E_g_ value from the onset of the IPCE spectrum as 1.84 eV, which is in good agreement with the bandgap value obtained from the UV-visible absorption band; hence, these supporting data validate our findings.

**Figure 4:**
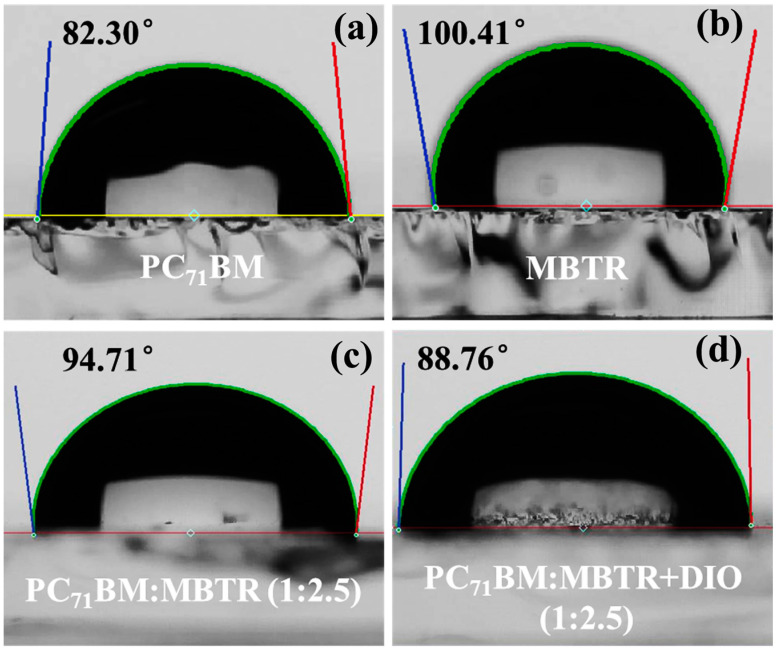
Contact angle measurements of (**a**) PC_71_BM; (**b**) MBTR; (**c**) PC_71_BM:MBTR (1:2.5); (**d**) PC_71_BM:MBTR (1:2.5) with DIO additive.

We additionally conducted a water contact angle analysis to explain the surface compositions and wettability of the active layer films with and without the DIO additive, as demonstrated in Figure 4. The water contact angles of the pure PC_71_BM and MBTR films were 82.30° and 100.41°, revealing the hydrophilic and hydrophobic top surfaces of both films, respectively [55]. The contact angle of the PC_71_BM:MBTR blend film processed with 3% DIO was approximately 88.76°, which was relatively lower than the contact angle of PC_71_BM:MBTR (94.71°), indicating the richness of the PC_71_BM content on the top of the blend surface. Therefore, an appreciative ohmic contact is formed with the metal cathode owing to the smaller contact angle, thus improving the performances of the fabricated solar devices [56].

### 2.4. Thin-Film Morphology

**Figure 5:**
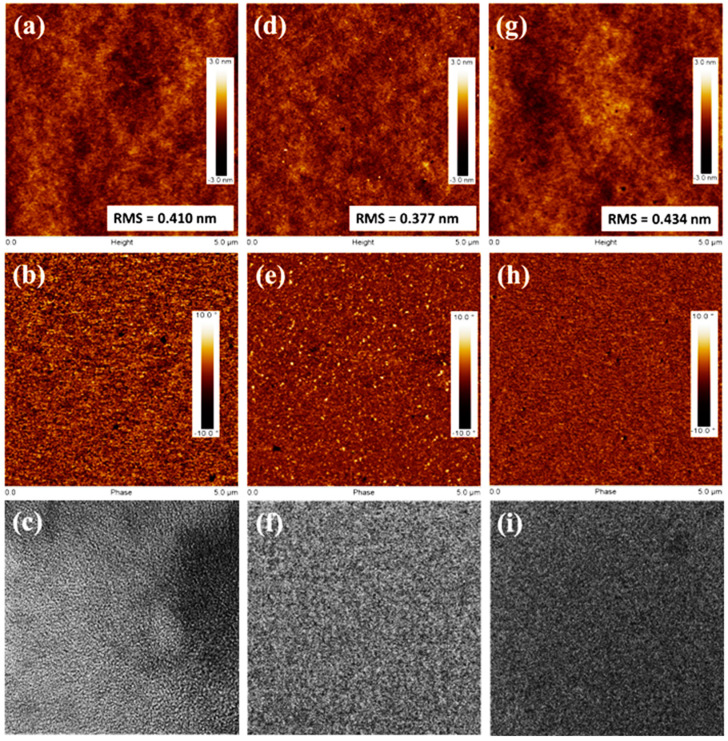
AFM height, phase, and TEM images of (**a**–**c**) MBTR:PC_71_BM (1:2.5); (**d**–**f**) MBTR:PC_71_BM (1:2.5) with DIO additive; (**g**–**i**) MBTR:PC_71_BM (1:3.0).

The surface morphology of the MBTR:PC_71_BM blend film acquired by atomic force microscopy (AFM) and transmission electron microscopy (TEM) are demonstrated in Figure 5. For efficient exciton separation and transport in BHJ OSCs, the active layer domain size should not be too small or too large compared with the exciton diffusion length of 15–20 nm [57]. The blend films of MBTR:PC_71_BM (1:2.5 and 1:3.0) cast with chlorobenzene showed decent phase-separated morphologies with root mean-squared (RMS) roughness values of 0.410 nm and 0.434 nm, respectively. Some irregularities were found on the surface, which may increase the R_S_ and adversely affect the V_OC_ and FF values. After processing with DIO (Figure 5d–e), more compatible fibrous domains were observed owing to the crystallinity features of the MBTR molecule, as confirmed by the DSC results. The average domain size was in the range of 15–20 nm, and the surface roughness was reduced from 0.410 to 0.377 nm, which is beneficial for carrier transport, resulting in higher photocurrent density (J_SC_) and FF values [47]. The TEM images of the MBTR:PC_71_BM (1:2.5) with DIO displayed a dense and notable intermixed network of D/A at the BHJ surface. Appropriate phase separation and average domain size with a controlled BHJ surface can mitigate charge recombination, thus scaling up exciton separation and their transport at the interface for enhanced J_SC_, V_OC_, and FF of the solar device [49].

## 3. Materials and Methods

All the materials and reagents employed in this study are high-purity grade and were used without further treatment. The 2,1,3-benzothiadiazole, 2-(tributylstannyl)thiophene, (E)-2-(3,5-dimethoxystyryl)-4,4,5,5-tetramethyl-1,3,2-dioxaborolane, and 5′-hexyl-2,2-bi–thiophene-5-boronic acid pinacol ester were acquired from Sigma Aldrich chemical company (St. Louis, MO, USA). The hydrobromic acid, bromine, and N-bromosuccinimide were purchased from Tokyo Chemical Industry (TCI) (Tokyo, Japan).

### 3.1. Synthesis of (E)-4-(5-Bromothiophen-2-yl)-7-(5-(3,5-dimethoxystyryl)thiophen-2-yl)–benzo[c][1,2,5]thiadiazole (Compound ***7***)

Compound **7** was synthesized by adding 1.23 g, 4.25 mmol of (E)-2-(3,5-dimethoxystyryl)-4,4,5,5-tetramethyl-1,3,2-dioxaborolane (6) to a degassed solution of monomer 5 (1.5 g, 3.27 mmol) in 100 mL of dry toluene. Immediately after the temperature of the hot plate exceeded 100 °C, a Pd(PPh_3_)_4_ catalyst at 5 mol% (0.185 g, 0.16 mmol) and 20 mL aqueous solution of K_2_CO_3_ (2M) were added and heated overnight at 120 °C with vigorous stirring. After the reaction, the substance was quenched with water, extracted with excess dichloromethane (DCM) and deionized (DI) water, saturated with a NaCl solution (4.4%), and dried over anhydrous Mg_2_SO_4_. The purification was performed on silica gel column chromatography using hexane:ethyl acetate in a 20:2 ratio by preparing a thick slurry of the crude product to obtain monomer 7 as a dark-brown solid (1.2 g, yield = 67%). ^1^H NMR (400 MHz, CDCl_3_, ppm): 8.10 (d, *J* = 3.4, Hz, 1H), 7.84–7.90 (m, 2H), 7.83 (s, 2H), 7.31 (d, *J* = 6.84 Hz, 1H), 7.20 (d, *J* = 3.92, 1H), 7.05 (s, 2H), 6.71 (d, *J* = 1.92 Hz, 1H), 6.47 (s, 1H), 3.90 (s, 6H). ^13^C NMR (100 MHz, CDCl_3_, δ_C_/ppm): 161.4, 152.7, 144.6, 141.1, 139.1, 138.3, 131.0, 129.5, 128.7, 127.9, 127.4, 126.4, 125.5, 125.2, 122.5, 114.9, 104.8, 100.6, 55.6. MS, m/z; calculated for [C_24_H_17_BrN_2_O_2_S_3_ +H]^+^: 542.5030, found 542.7250.

### 3.2. Synthesis of (E)-4-(5-(3,5-Dimethoxystyryl)thiophen-2-yl)-7-(5″-hexyl-[2,2′:5′,2″-terthiophen]-5-yl)benzo[c][1,2,5]thiadiazole (MBTR)

Under nitrogen flow, products 7 (0.700 g, 1.30 mmol) and 8 (0.587 g, 1.56 mmol) were mixed in 60 mL of dry toluene and stirred at 120 °C to obtain a homogenous solution. Thereafter, 3 mol% Pd(PP_3_)_4_ catalyst (0.045 g, 0.039 mmol) and an aqueous 2M solution of K_2_CO_3_ were added stepwise to the reaction mixture with continuous heating at 120 °C for 24 h. The resulting mixture was cooled and poured into a separating funnel containing 50 mL of DCM. The organic phase was separated by adding DI water, collected, and dried by portion-wise addition of Mg_2_SO_4_. The preabsorbed product on silica gel beads (400–650 mesh) was added to a silica-packed column and purified using hexane:DCM (10:2). Further, the dried crude was recrystallized through ethyl alcohol and hexane to obtain MBTR as a dark solid (0.540 g, yield = 58%). ^1^H NMR (600 MHz, CDCl_3_, ppm): 8.06 (t, *J* = 4.44 Hz, 4.8 Hz, 2H), 7.86 (d, *J* = 2.4 Hz, 2H), 7.25 (d, *J* = 4.8 Hz, 1H), 7.23 (s, 1H), 7.18 (d, *J* = 4.5 Hz, 1H), 7.16 (d, *J* = 4.5 Hz, 1H), 7.04 ((d, *J* = 4.8 Hz, 1H), 7.01 (t, *J* = 3.48 Hz, 2H), 6.98 (s, 1H), 6.70 (d, *J* = 4.08 Hz, 1H), 6.67 (d, *J* = 2.4 Hz, 1H), 6.41 (s, 1H), 3.85 (s, 6H), 2.81 (t, *J* = 8.94 Hz, 2H), 1.31–1.34 (m, 4H), 1.68–1.73 (m, 4H), 0.89 (t, *J* = 8.58 Hz, 3H). MS: m/z calc. for [C_38_H_34_N_2_O_2_S_5_ + H]^+^: 712.0138, found: 712.0175. FTIR (KBr, Pellet): 3064, 3020, 2977, 2957, 2926, 2851, 1591, 1533, 1487, 1450, 1348, 1293, 1207, 1152, 1112, 1065, 948, 877, 854, 837, 795, 677, 638, 520.

### 3.3. Characterization

The MBTR molecular structure was elucidated by ^1^HNMR, FTIR (JASCO, 4100-FTIR, Tokyo, Japan), and mass (TQ-S, XEVO) spectroscopy measurements. A TGA Ta instrument (TGA Q50) and DSC instrument (Shimadzu, DSC-60, Kyoto, Japan) at a constant heating rate of 5 °C/min under a nitrogen atmosphere were utilized to determine the thermal stability and crystal nature of MBTR. The solution and thin-film absorbance of the MBTR were recorded with a JASCO ultraviolet-visible (UV–vis) spectrophotometer (V–670). Fluorescence emission spectra and time-resolved photoluminescence (TRPL) measurements in solution as well as for the thin film and blend films were carried out on a 4th generation Horiba Fluorolog-QM (Kyoto, Japan) spectrofluorometer at the future energy convergence core (FECC) center, Jeonbuk National University. The CV curves were determined with a three-electrode system with Ossila potentiostat containing an Ag/AgCl^−^, a Pt wire, and a glassy carbon electrode used as the reference, counter, and working electrodes, respectively. The electrolyte solution for the CV measurement was prepared with 0.1 M tetrabutylammonium hexafluorophosphate (Bu_4_NPF_6_) solution in dry acetonitrile. The contact angle of the active layer film was investigated via a contact angle goniometer instrument (Ossila, L2004A10, Sheffield, UK) using water as the reference solvent. The photovoltaic properties of the solar devices were evaluated by measuring the J–V curves on a solar simulator device (Newport, 94022A, Irvine, CA, USA) under a light source of 100 mW/cm^2^ at AM 1.5 radiation. The quantum efficiency of the BHJ OSCs was measured using an IPCE Spectrometer. The surface morphology of the MBRT:PC_71_BM active layer was examined with an atomic force microscope system (Nanoscope V multimode 8 AFM) and field-effect transmission electron microscopy (FE–TEM) equipment (JEM–220FS (HT)).

### 3.4. Device Fabrication of BHJ OSCs

The OSCs were fabricated with the conventional ITO/PEDOT:PSS/MBTR:PC_71_BM(*w/w*)/Au structure. Transparent ITO glass was critically cleaned in an ultrasonic bath using 2 mL hellmanex III solution in 200 mL hot water for 5 min. Next, the substrates were sequentially cleaned with DI water, acetone, and ethanol each for 10 min and dried in a vacuum oven. Further, the cleaned substrates were placed in UV–ozone equipment and exposed to UV–ozone for surface treatment for 15 min to remove the surface impurities and improve adhesion properties. We used solar-grade PEDOT:PSS (HTL Solar) for deposition of the buffer layer, which was filtered through a 0.45 μm polytetrafluoroethylene (PTFE) filter using a rubber-free syringe. The freshly prepared PEDOT:PSS solution was spin coated on the cleaned substrates at the rate of 4000 RPM for 30 s and annealed over a previously heated hot plate. Varying blend ratio (*w/w*) solutions were prepared by mixing MBTR and PC_71_BM in absolute chlorobenzene using a constant concentration of 30 mg/mL. The blend solution with 3% DIO additive for optimized devices was prepared in a volume ratio of chlorobenzene/DIO (97/3 *v*/*v*%) with a fixed active material concentration of 30 mg/mL. Next, the blend solution with and without DIO was spin-coated on ITO/PEDOT:PSS at a rate of 3000 RPM for 40 s and immediately baked at 80 °C for 10 min. Finally, the ITO/PEDOT:PSS/MBTR:PC_71_BM-deposited substrate was placed in a shadow mask, and a metal electrode layer of Au (gold) was thermally evaporated under a high vacuum of 1 × 10^−6^ Torr. The BHJ PV devices were fabricated at ambient temperature with an active area of 0.12 cm^2^.

## 4. Conclusions

This work demonstrates the synthesis of a low-bandgap BTD-based novel small-molecule donor (MBTR) linked through the thiophene π-bridge and end-capped with different donor units. The MBTR displayed a strong absorption band in the UV-visible region and an optical bandgap of 1.86 eV. The deep HOMO-LUMO levels and low energy offset between the MBTR and PC_71_BM promote the separation of the photogenerated excitons and charge transport at the BHJ interface. We fabricated organic solar devices with and without a DIO additive and examined the potential of the MBTR donor in combination with a fullerene acceptor. The solar cell based on the MBTR:PC_71_BM (1:2.5 *w/w*%) with DIO realized a high PCE of 8.76% with J_SC_ of 13.78 mA/cm^2^, V_OC_ of 1.021 V, and FF of 62.3% compared to the PCE of 7.05% without the additive. The high PCE with improved PV parameters is attributed to the appropriate phase separation in the active layer, charge transport, and low energy loss in BHJ OSCs. This work suggests that suitable choices of the donor and acceptor units in designing small organic molecules can afford more competent donors for economically feasible OSCs.

## Figures and Tables

**Table 1:** Thermal, optical, and electrochemical results of the synthesized donor small molecule.

Molecule	T_d_ (°C)	λ_max_ ^a^ (nm)	λ_max_ ^b^ (nm)	E_ox_^onset^ (V)	E_red_^onset^ (V)	HOMO ^c^ (eV)	LUMO ^d^ (eV)	E_g_^cv^ (eV)	E_g_^opt^ (eV)
MBTR	430	404, 515	699	+0.62	−1.20	−5.42	−3.60	1.81	1.86

^a^ Absorbance of MBTR in thin film, ^b^ PL emission spectra of MBTR in thin film, ^c^ HOMO and ^d^ LUMO energy levels.

**Table 2:** Photovoltaic results of the synthesized donor small molecule.

	(*w/w* Ratio)	V_oc_ (V)	J_sc_ (mA/cm^2^)	FF (%)	R_s_ (Ω cm^2^)	R_sh_ (Ω cm^2^)	PCE (%)	J_sc_^IPCE^ (mA/cm^2^)
MBTR	1:1.5	0.910	10.36	45.0	1.62	22.43	4.24	9.88
	1:2.0	0.926	11.01	50.5	3.02	42.31	5.15	10.38
	1:2.5	0.943	12.63	59.2	1.59	60.50	7.05	12.29
	1:2.5 (3% DIO)	1.021	13.78	62.3	1.20	69.78	8.76	13.51
	1:3.0	0.947	12.15	59.3	1.47	57.71	6.82	11.70
	1:3.5	0.859	9.97	46.1	2.39	26.91	3.95	9.38

## Data Availability

Not applicable.

## Supplementary Materials

The following supporting information can be downloaded at: https://www.mdpi.com/article/10.3390/molecules28041538/s1, S1.1: Synthesis of intermediate 4,7-dibromobenzothiadiazole (2); S1.2: Synthesis of monomer 4,7-dithienyl-2,1-3-benzothiadiazole (4); S1.3: Synthesis of 4,7-bis(5-bromothiophen-2-yl)benzo[c][1,2,5]thiadiazole (5); Figure S1: ^1^H NMR spectrum of the MBTR molecule.; Figure S2: Mass spectroscopy plots of (a) intermediate 7 and (b) MBTR molecule; (c) FTIR spectra of MBTR molecule.; Figure S3: 4th quadrant I-V characteristics of BHJ OSCs with different *w/w*% ratio of MBTR:PC_71_BM; (a) MBTR:PC_71_BM (1:2.0), (b) MBTR:PC_71_BM (1:2.5), (c) MBTR:PC_71_BM (1:2.5) with DIO and (d) MBTR:PC_71_BM (1:3.0). S.2: FF of BHJ OSCs [58,59,60].

## Sample Availability

Samples of the compounds are available from the authors by request.
