# Supplementary material for: Linear-Shaped Low-Bandgap Asymmetric Conjugated Donor Molecule for Fabrication of Bulk Heterojunction Small-Molecule Organic Solar Cells
Source: Molecules. 2023 Feb 5;28(4):1538. doi: 10.3390/molecules28041538 (PMC9964613; doi:10.3390/molecules28041538)
Supplement: Supplementary file 1 [file molecules-28-01538-s001.zip › molecules-2197980-supplementary.pdf]

Supporting Information

Linear-Shaped Low-Bandgap Asymmetric Conjugated Donor Molecule for Fabrication of Bulk Heterojunction Small-Molecule Organic Solar Cells

Abdullah [1], Sei-Jin Lee [1,*], Jong Bae Park [1], Yang Soo Kim [1], Hyung-Shik Shin [2], Ashique Kotta [3,4], Qamar Tabrez Siddiqui [3], Youn-Sik Lee [3,*], Hyung-Kee Seo [3,4,*]

[1]Korea Basic Science Institute (KBSI), Jeonju Center, Jeonju, 54896, Republic of Korea

[2]Korea Basic Science Institute (KBSI), Daejeon, 34133, Republic of Korea

[3] School of Chemical Engineering, Jeonbuk National University, Jeonju, 54896, Republic of Korea

[4] Future Energy Convergence Core Center, Jeonbuk National University, Jeonju, 54896, Republic of Korea

* Correspondence: lsj@kbsi.re.kr (S.-J.L.); hkseo@jbnu.ac.kr (H.-K.S.); yosklear@jbnu.ac.kr (Y.-S.L.);
Fax: +82-63-270-4097 (H.-K.S.)

*Correspondence: lsj@kbsi.re.kr (S.-J.L.); hkseo@jbnu.ac.kr (H.-K.S.); yosklear@jbnu.ac.kr (Y.-S.L.), Fax: +82-63-270-4097 (H.-K.S.)

S1.1: Synthesis of intermediate 4,7-dibromobenzothiadiazole (2)

In a 250 ml two-neck flask, 2,1,3-benzothiadiazole (1) (10 g, 73.44 mmol) and 19.87 ml (13.34 g, 164.88 mmol) of 48% HBr were transferred and degassed for 10 min, followed by slow addition of 11.13 g of Br_2 (34.62 ml, 216.65 mmol) at 150 °C. Immediately after adding Br_2, the reaction mixture starts precipitating, which is significantly assisted by further addition of 40 ml HBr and heating at the same temperature for the next 2 h. The reaction mixture was then filtered and treated with DI water several times to obtain an off-white precipitate. To remove the excess bromine from the crude, an aqueous sodium thiosulfate (10%) solution was used until the neutral pH point. The dried crude was then transferred to a beaker containing 200 ml ethyl alcohol and allowed to crystallize at room temperature. White needle-like crystals (20.8 g, yield= 96%) were obtained from the recrystallization process in a mixed solution of ethyl alcohol and chloroform. 1H NMR (400 MHz, $CDCl_3$, ppm): 7.75 (s, 2H). ^{13}C NMR (100 MHz, $CDCl_3$, ppm)): 153.1, 132.4, 113.9. MS, m/z; calculated for $[C_6H_2Br2N_2S]^+$:293.973, found 293.971.

S1.2: Synthesis of monomer 4,7-dithienyl-2,1-3-benzothiadiazole (4)

Intermediate 2 (3.0 g, 10.21 mmol) was dissolved in dry tetrahydrofuran (THF; 120 ml) and purged with nitrogen for 10 min before adding 2-(tributlstannyl)thiophene (11.43 g, 30.63 mmol), followed by 3 mol% $PdCl_2(PPh_3)_2$. The resultant reaction mixture was heated to reflux temperature for 24 h. The cooled reaction mixture was then poured into a separating funnel and rinsed with dichloromethane (DCM) and DI water multiple times. The obtained organic layer was transferred to a beaker and dried with portion-wise addition of anhydrous Mg_2SO_4 before concentrating with a rotary evaporator. The obtained product was preabsorbed on silica gel to prepare a slurry and purified by the column chromatography technique, where hexane and ethyl acetate (10:1) were used as the eluents. The chromatograms were collected and dried to obtain the pure product (2.30 g, yield=75%) as shiny red crystals. ^1H NMR (400 MHz, $CDCl_3$, ppm):7.91 (dd, 2H), 7.67 (s, 2H), 7.25 (dd, 2H), 7.01 (m, 2H). ^{13}C NMR (100 MHz, $CDCl_3$, ppm): 152.7, 139.1, 128.2, 127.4, 126.9, 126.5, 125.7. MS, m/z; calculated for $[C_{14}H_8N_2S_3]^+$:299.985, found 299.984.

S1.3: Synthesis of 4,7-bis(5-bromothiophen-2-yl)benzo[c][1,2,5]thiadiazole (5)

Compound 5 was synthesized under dark conditions by dissolving monomer 4 (2.5 g, 8.32 mmol) in 80 ml of absolute $CHCl_3$ at 0 °C under an inert atmosphere. After complete mixing, about 3.70 g (20.8 mmol)

of NBS was added, and the reaction mixture was stirred overnight. The reaction was quenched with a small volume of DI water, and the red precipitate was filtered off and washed with excess water repeatedly. Then dry crude was first dissolved in a small portion of ethyl alcohol and then recrystallized from a solution of ethyl alcohol and chloroform to obtain dark red crystals (2.17 g, yield= 57%). ^{1}H NMR (400 MHz, $CDCl_3$, ppm): 7.81 (d, J = 4.4, Hz, 2H), 7.79 (s, 2H), 7.16 (d, 3.92 Hz, 2H). ^{13}C NMR (100 MHz, $CDCl_3$, ppm): 154.6, 138.9, 127.6, 127.1, 126.7, 125.2, 124.9. MS, m/z; calculated for $[C_{14}H_6Br_2N_2S_3 + H]^+$:458.803, found 458.807.

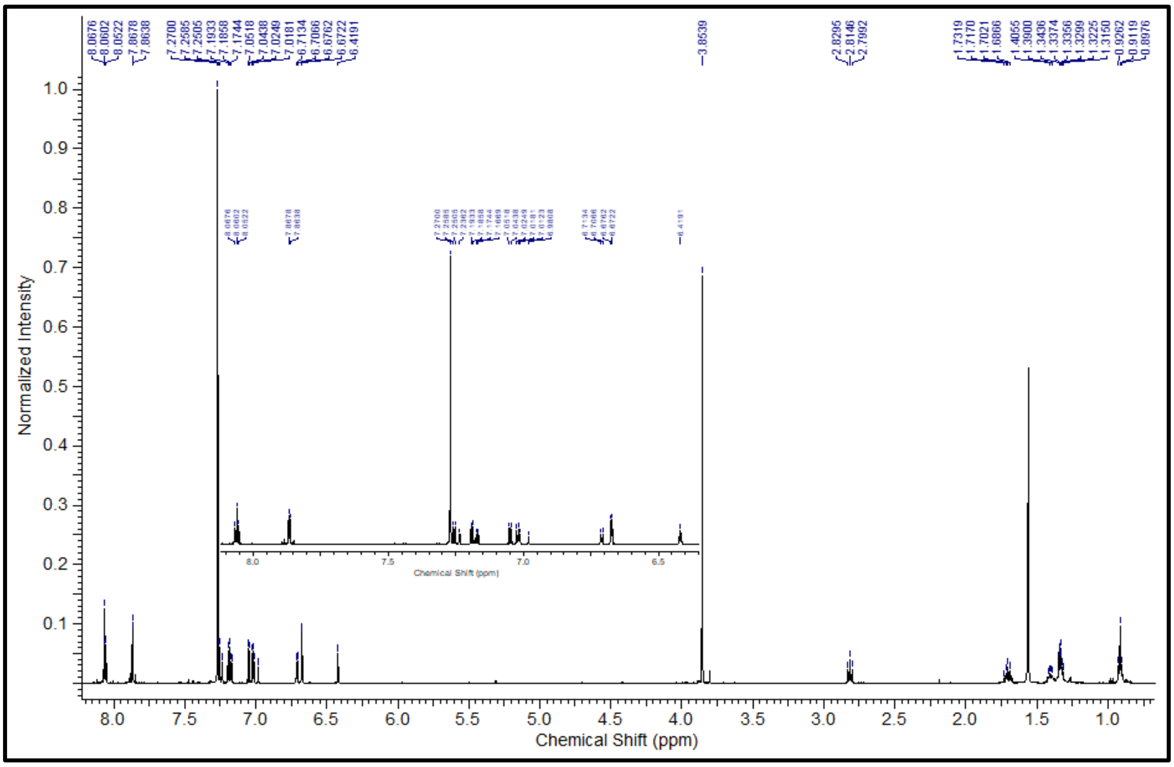

Figure S1. ^{1}H NMR spectrum of the MBTR molecule.

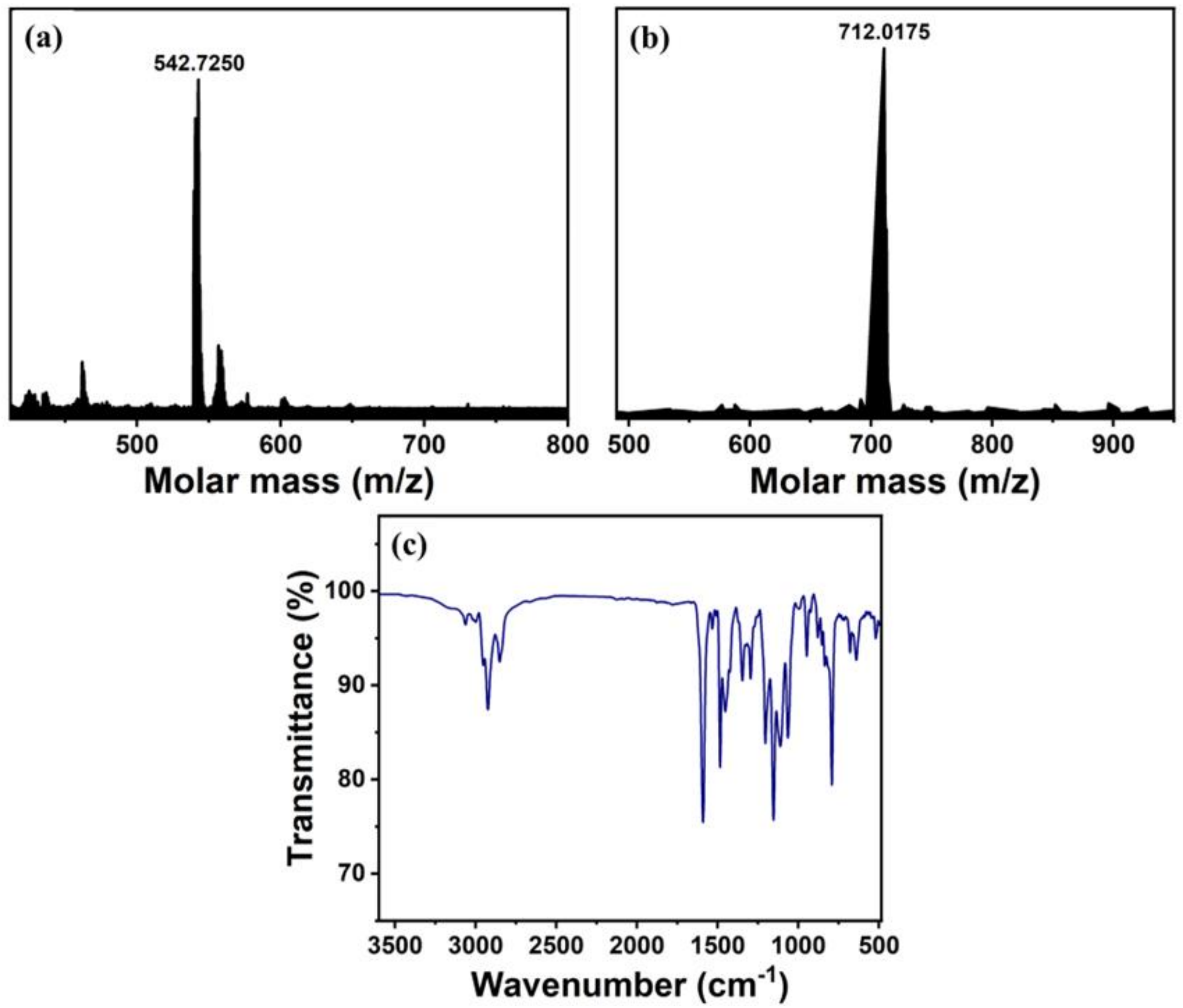

Figure S2. Mass spectroscopy plots of (a) intermediate 7 and (b) MBTR molecule; (c) FTIR spectra of MBTR molecule.

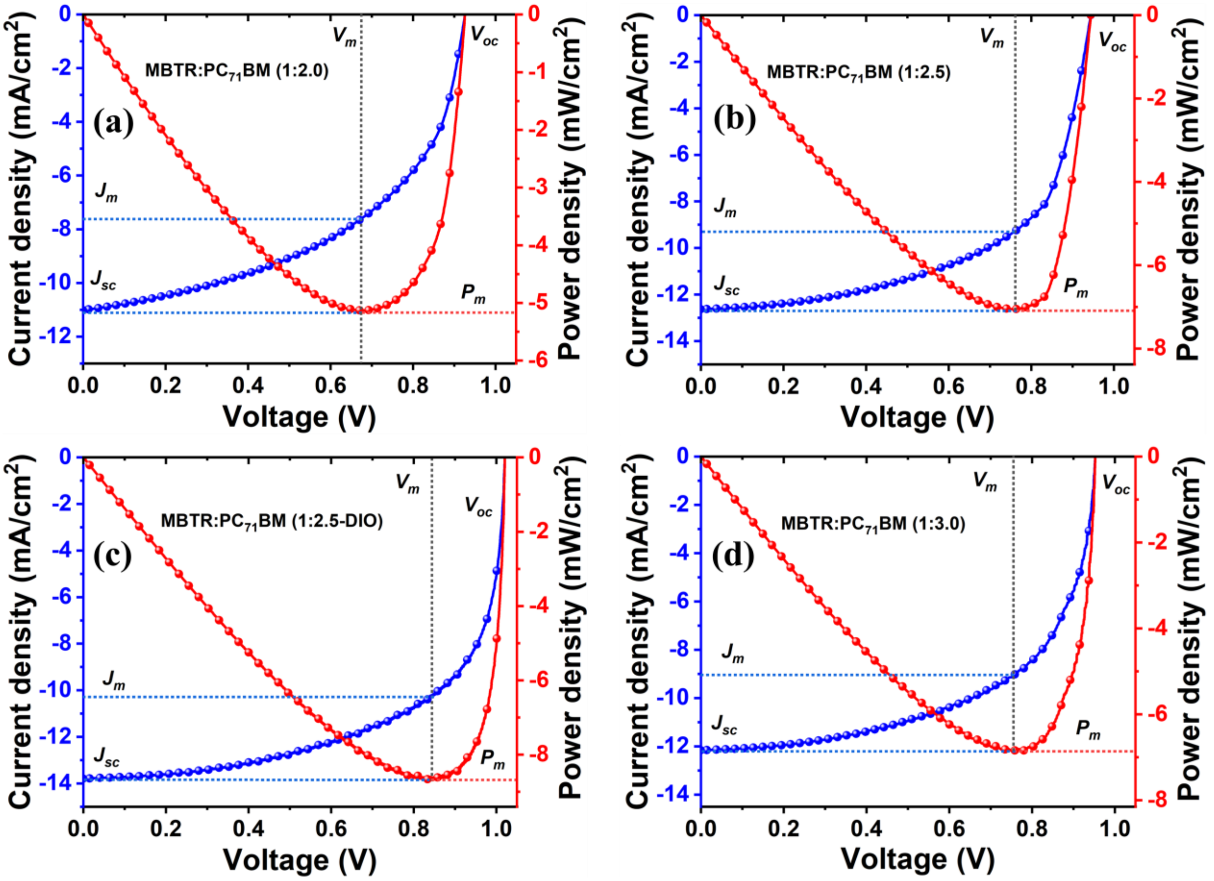

Figure S3. 4th quadrant I-V characteristics of BHJ OSCs with different w/w% ratio of MBTR:PC_{71}BM; (a) MBTR:PC_{71}BM (1:2.0), (b) MBTR:PC_{71}BM (1:2.5), (c) MBTR:PC_{71}BM (1:2.5) with DIO and (d) MBTR:PC_{71}BM (1:3.0).

S.2 FF of BHJ OSCs

The accuracy of FF for organic devices is investigated by the fourth quadrant I-V characteristics of MBTR:PC_{71}BM with the varying donor: acceptor (w/w% ratio) content under the illumination of 1.5 A.M. at 100 mW/cm^2. FF is characterized as maximum electrical power (P_m) from the solar device at the maximum voltage (V_m) and current (J_m) associated to the product of V_{oc} and J_{sc} [1-3]. The FF is calculated from the expression:

$$\mathrm{FF} = \frac{\mathrm{V_m\,J_m}}{\mathrm{V_{oc}\,J_{sc}}} \times 100$$

where $P_m = V_m J_m$

The PCE can be expressed by the following equation:

$$PCE = \frac{FF\,V_{oc}\,J_{sc}}{P_{light}}$$

The P_m is calculated with the help of the fourth quadrant I-V curve using the equation as 5.15 mW/cm^2, 7.05 mW/cm^2, 8.77 mW/cm^2 and 6.82 mW/cm^2 for MBTR:PC_{71}BM (1:2.0), MBTR:PC_{71}BM (1:2.5), MBTR:PC_{71}BM (1:2.5) with DIO and MBTR:PC_{71}BM (1:3.0).

References

1. Lucian, I.; Ionut, E.; Sorina, I.; Veta, G.; Cezar, T.; Cristina, B.; Todor, L.M.; Vlad, A.A.; Marin, M.G.; Stefan, A. Effects of Proton Irradiation on the Spectral Performance of Photovoltaic Cells Based on CdS/CdTe Thin Films. Chalcogenide Letters 2010, 7(8), 2010, 521 -530.
2. Adrian, R.; Sorina, I.; Veta, G.; Cristina, B.; Vlad, A.A.; Gvido, B.; Lucian, I.; Simona, C.; Mihaela, G.; Stefan A. The Influence of LiF Layer and ZnO Nanoparticles Addings on the Performances of Flexible Photovoltaic Cells Based on Polymer Blends. Digest Journal of Nanomaterials and Biostructures 2011, 6 (3), 1141-1148.
3. Stefan, A.; Sorina, I.; Laura, H.; Vlad, A.A.; Mihaela, G. A Critical Review of Photovoltaic Cells Based on Organic Monomeric and Polymeric Thin Film Heterojunctions. Thin Solid Films 2017, 642, 219–231.
